# Mechanical Enhancement for Longevity of Glass Ionomer Cement Using Cellulose Nanocrystals: A Study on Microhardness and Wear Resistance

**DOI:** 10.1155/ijod/6922800

**Published:** 2026-09-23

**Authors:** Kiran Manjunath, Vinod Jathanna, Kishore Ginjupalli, Shreya Hegde

**Affiliations:** ^1^ Department of Conservative Dentistry and Endodontics, Manipal College of Dental Sciences Mangalore, Manipal Academy of Higher Education, Manipal, India, manipal.edu; ^2^ Department of Dental Materials, Manipal College of Dental Sciences, Manipal Academy of Higher Education, Manipal, India, manipal.edu

**Keywords:** cellulose nanocrystals, dental biomaterials, glass ionomer cement, nanocomposites, surface microhardness, wear resistance

## Abstract

**Background:**

Glass ionomer cement (GIC) has found extensive application in the field of restorative dentistry due to its chemical adherence to tooth structure and fluoride releasing characteristics. Nevertheless, its clinical use in load bearing, posterior restorations is constrained by poor surface microhardness and wear resistance. Cellulose nanocrystals (CNCs) are bio‐derived nanomaterials which have high bond strength making them potential reinforcing agents in dental cements.

**Objective:**

To evaluate the surface microhardness and wear resistance of GIC incorporated with CNCs at concentrations of 0.2%, 0.5%, and 1% in comparison to an unmodified control.

**Methods:**

An in vitro experimental study was carried out on four groups (*n* = 12 per group): control (unmodified GIC), G1 (GIC + 0.2% CNCs), G2 (GIC + 0.5% CNCs), and G3 (GIC + 1% CNCs). Standardized rectangular specimens were prepared. The surface microhardness was measured by the use of a Vickers hardness tester and the wear resistance was evaluated on a pin‐on‐disc wear testing apparatus.

**Results:**

The highest mean surface microhardness was seen in G3 (208.1 ± 0.56), followed by G2 (157.4 ± 0.32), G1 (133.3 ± 0.37), and the lowest measurements were seen in the control group (98.3 ± 0.32 Vickers hardness number [VHN]). Mean wear resistance was highest in the control group (48.3 ± 0.31), followed by G2 (30.2 ± 0.28), G1 (30.1 ± 0.17), and the lowest in G3 (16.9 ± 0.26 VHN). The differences among the groups were statistically significant (*p* < 0.001).

**Conclusion:**

Incorporation of CNCs significantly improved the surface microhardness of restorative GIC in a concentration‐dependent manner. However, higher CNCs concentrations were associated with reduced wear resistance. Further long‐term studies are needed to determine the optimal CNCs concentration for clinical applications.

## 1. Introduction

Glass ionomer cement (GIC) is a widely used restorative material introduced in the 1970s. The extensive use of GIC is attributed to its chemical adhesion to enamel and dentin, fluoride release, biocompatibility, and thermal compatibility with dental tissues [1, 2]. These features favor its use in minimally invasive dentistry such as atraumatic restorative treatment (ART), where tooth preservation is vital. Additionally, clinical data supports the use of high‐viscosity GIC restorations in contemporary restorative therapy as a substitute for silver amalgam [3, 4]. In spite of these benefits, traditional GIC has natural mechanical constraints, such as poor fracture toughness, wear resistance and surface microhardness, which limit its application in stress‐bearing regions [5]. These shortcomings have prompted comprehensive research efforts on material modification approaches to enhance their structural performance without interfering with their biological characteristics. Fiber reinforcement is one of such strategies that were studied extensively. Initial research revealed that addition of cellulosic fibers in GIC enhanced compressive strength, wear resistance and adhesion without impairing satisfactory physicochemical attributes [6]. Later studies showed that mechanically processed cellulose fibers increased the compressive and tensile strength, but the gains were contingent on the level of concentration and dispersion of the fibers in the matrix. Nonetheless, macro and micro fibers tend to have low reinforcing capacity because of weak interfacial bonding and discontinuous distribution [7, 8].

The development of nanotechnology has rendered the usage of nanocellulose, or cellulose nanocrystals (CNCs), as a novel renewable nanomaterial [9]. There is systematic evidence that nanocellulose has a consistent positive effect on the mechanical performance and functional versatility of dental materials and does not affect or deteriorate biocompatibility [10]. Cellulose nanocrystals‐reinforced GIC has been experimentally studied and found to improve the mechanical characteristics [2]. Moradian et al. [1] showed that incorporating bacterial cellulose nanocrystals (BCNCs) into resin‐modified GIC improved its mechanical properties, with 1 weight percentage (wt%) BCNC increasing compressive and diametral tensile strength by approximately 23% and the elastic modulus by 44%. Silva et al. [11, 12] across multiple studies and Nishimura et al. [13] reported an increase in compressive strength, abrasion resistance, and bond strength to the dental structure after the addition of cellulose fibers. Mesquita et al. [14] demonstrated that cellulose nano whiskers can reinforce chitosan‐based nanocomposites through strong polymer–nanofiller interactions and homogeneous nano whisker distribution. Similarly, Rini et al. [15] reported that incorporating rice husk‐derived nanocrystalline cellulose (NCC) into GIC improved hardness and shear bond strength, with the greatest improvements observed at 1% NCC, although compressive and flexural strength were not significantly affected. Likewise, other studies indicate increased microhardness, tensile strength, and general structural integrity of GIC by cellulose nanomaterials without negatively impacting on the release of fluoride and handling characteristics [13, 16]. Recent strategies for improving the mechanical performance of GIC have focused on biopolymer modification, functionalized glass fibers, and oxidized natural polymers. Alsunbul et al. [17] and Khan et al. [18] demonstrated that oxidized biopolymers improved particle bonding and enhanced the physical and mechanical properties of GIC. Similarly, functionalized micron‐sized glass fibers have shown synergistic reinforcement effects, improving strength and durability [19].

Despite the accumulating evidence supporting CNCs reinforcement, critical gaps remain. A majority of the studies [9, 11, 12] have assessed general mechanical properties like compressive and tensile stress with less attention given to clinically relevant parameters like surface microhardness and resistance. Furthermore, there is insufficient evidence regarding how varying concentrations of CNCs influence these properties, making it difficult to identify optimal formulations for consistent clinical outcomes. These gaps are particularly relevant from a public health perspective, especially in resource‐limited and community‐based settings where GIC is commonly used in minimally invasive approaches such as ART. The incorporation of sustainable nanomaterials such as cellulose nanocrystals to improve surface microhardness and wear resistance may help enhance restoration longevity and potentially reduce the need for follow‐up visits, thereby supporting more efficient and accessible oral healthcare delivery. In this context, the present study aimed to evaluate the wear resistance and surface microhardness of GIC incorporated with nanocellulose crystals in various concentrations. The objectives were as follows (i) to evaluate and compare the surface microhardness of GIC incorporated with 0.2% CNCs (G1), 0.5% CNCs (G2), and 1% CNCs (G3) against an unmodified GIC control. (ii) To evaluate and compare the wear resistance of GIC incorporated with 0.2%, 0.5%, and 1% CNCs against an unmodified GIC control. (iii) To determine the relationship between CNCs concentration and changes in the mechanical properties of GIC. The study hypothesized that the incorporation of cellulose nanocrystals improved the surface microhardness and wear resistance of conventional restorative GIC.

## 2. Materials and Methods

### 2.1. Sample Collection and Preparation

This in vitro experimental study was conducted following approval from the Institutional Ethics Committee of Manipal College of Dental Sciences, Mangalore (IEC Protocol No. 24107). The sample size was calculated based on previous studies [9, 16] with surface microhardness as the primary outcome variable. An expected standard deviation (*σ*) of 18.32 was adopted from the reference study [16]. Using a significance level of 0.05, a statistical power of 80% and a minimum clinically significant difference (*d*) of 3 units, the required sample size was estimated using the formula:

n=Z12−α/+Z1−β2σ2d2.

Based on this calculation, the required sample size was estimated to be 12 specimens per group, resulting in a total of 48 specimens for the four study groups. Conventional restorative type II GIC (GC Gold Label 2, GC Corporation, Tokyo, Japan) was modified using cellulose nanocrystals. Cellulose nanocrystals (CNCs) were procured from Vedayukt India Pvt. Ltd. (Batch No. 60258) and used as received. According to the manufacturer’s specifications, the CNCs were supplied as a dry powder comprising rod‐shaped nanocrystals with widths ranging from 10 to 20 nm and lengths ranging from 300 to 900 nm. The physicochemical properties, including particle dimensions and morphology, were obtained from the manufacturer’s characterization data. No additional surface characterization was performed prior to the experimental use. The cellulose nanocrystals were incorporated into the GIC powder according to the designated concentration. To ensure homogeneous distribution and minimize particle agglomeration, the CNCs–GIC powder mixture was subjected to sonication using a bath sonicator operating at 80 W for 2 min at room temperature. The modified powder was then mixed with the GIC liquid according to the manufacturer’s recommended powder‐to‐liquid ratio of 1:1. Mixing was performed manually using a cement spatula on a mixing pad until a homogeneous consistency was obtained. The control group was prepared similarly without the addition of CNCs. Acrylic molds measuring 4 mm × 8 mm × 3 mm were fabricated, and the GIC after manipulation was placed into these molds. Standardized rectangular test specimens (ts) were prepared. Four experimental groups were established: control (unmodified GIC), G1 (GIC + 0.2% CNCs), G2 (GIC + 0.5% CNCs), and G3 (GIC + 1% CNCs). Each group consisted of 12 specimens.

All specimens were subjected to standardized wet polishing using sequential 320‐, 400‐, and 600‐grit silicon carbide abrasive papers [20]. Following polishing, the specimens were stored in distilled water at 37°C for 48 h before testing. Separate sets of specimens were prepared for surface microhardness testing and wear testing to eliminate any potential influence of indentation on wear measurements. Surface microhardness was assessed using a Digital Micro Hardness Tester (ASI‐MHT‐1000AT, Asian Test Equipments, India) equipped with a Vickers diamond indenter. A load of 300 gf was applied for 15 s for each indentation. Three indentations were made on each specimen at different locations while maintaining adequate spacing between the indentations and specimen margins. The mean of the three readings was recorded as the final Vickers hardness number (VHN) for statistical analysis.

**Figure 1:**
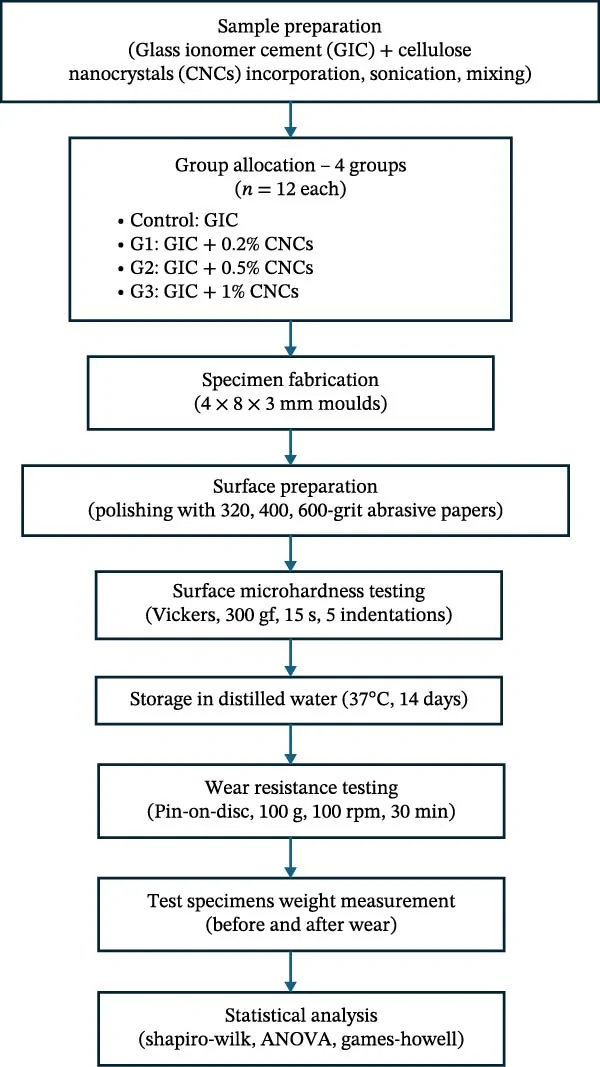
Schematic flowchart illustrating the experimental methodology for preparation and evaluation of CNCs‐modified glass ionomer cement.

**Figure 2:**
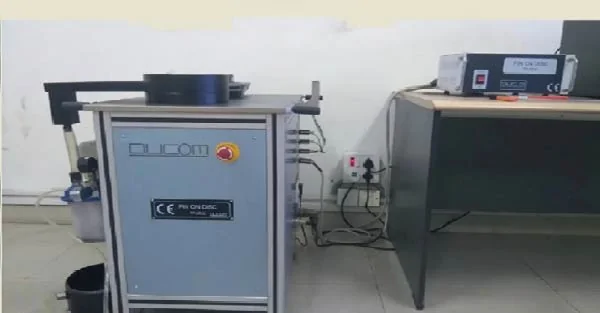
Image of pin‐on‐disc apparatus.

Wear resistance was assessed using a pin‐on‐disc apparatus [21] with the following parameters: alumina cylinder pin with a spherical end (spherical end diameter: 200 mm), applied load of 5 N, rotational frequency of 2 Hz, wear track radius of 2.5 mm, sliding speed of 31.4 mm/s, distilled water as lubricant, and a total of 7500 cycles. Prior to testing, each specimen was weighed using a precision analytical balance to obtain its initial weight. Following completion of the wear test, the specimens were cleaned, dried, and reweighed. Wear resistance was determined by calculating the difference between the initial and final specimen weights. The flow chart of the methodology is illustrated in Figure 1 and the image of the pin‐on‐disc apparatus in Figure 2.

### 2.2. Statistical Analysis

Statistical analysis was performed using Jamovi software (version 2.7.15). The distribution of data was evaluated for normality using the Shapiro–Wilk test. Intergroup comparisons were conducted using one‐way analysis of variance (ANOVA), and pairwise differences were further examined with the Games–Howell post hoc test. Statistical significance was set at a *p*‐value of less than 0.05.

## 3. Results

**Figure 3:**
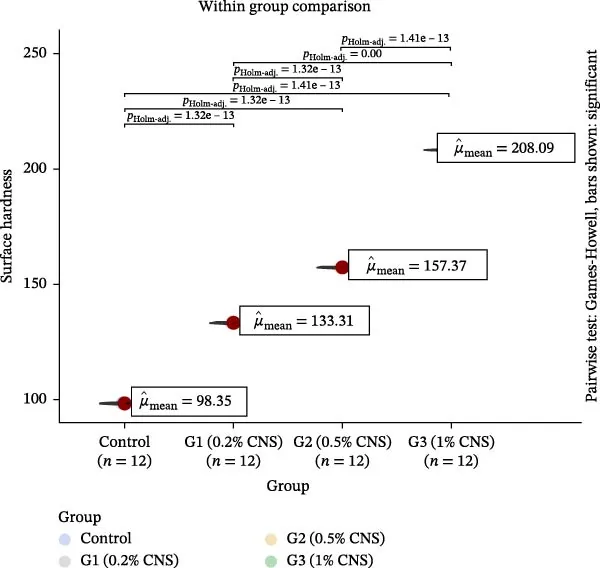
Distribution of surface microhardness across study groups using violin plot analysis.

Data was found to be normally distributed for all outcome variables (*p* > 0.05). Mean surface microhardness increased progressively from the control group (98.3 ± 0.32) to G1 (133.3 ± 0.37), G2 (157.4 ± 0.32), and G3 (208.1 ± 0.56). A statistically significant difference in surface microhardness was observed across the groups (*F* = 131,331, *p* < 0.001) (Tables 1 and 2). Post hoc comparisons indicated that all pairwise differences were statistically significant (all *p* < 0.001), confirming a dose‐dependent increase in surface microhardness (Table 3). The distribution of surface microhardness values is depicted in Figure 3. The modified GIC exhibited a working time comparable to conventional GIC, with no observable change in the final setting time.

**Table 1:** Comparison of wear resistance and surface microhardness between study groups.

Parameter	Group	*N*	Mean ± SD	Range (Min–Max)	Median (25^th^–75^th^ percentile)	*F*	*p*‐Value	Post hoc test
Wear resistance	Control	12	48.3 ± 0.310	47.6–48.8	48.3 (48.1–48.5)	22,933	**<0.001** ^∗^	G1 vs. G2: NS; others significant
	G1 (0.2% CNCs)	12	30.1 ± 0.167	29.9–30.4	30.1 (29.9–30.2)			
	G2 (0.5% CNCs)	12	30.2 ± 0.281	29.8–30.8	30.2 (30.0–30.3)			
	G3 (1% CNCs)	12	16.9 ± 0.259	16.4–17.4	16.9 (16.7–17.0)			
Surface microhardness	Control	12	98.3 ± 0.323	97.8–98.9	98.3 (98.1–98.6)	131,331	**<0.001** ^∗^	All sub groups are significant
	G1 (0.2% CNCs)	12	133.3 ± 0.370	132.6–133.8	133.3 (133.1–133.6)			
	G2 (0.5% CNCs)	12	157.4 ± 0.323	156.8–157.8	157.4 (157.2–157.6)			
	G3 (1% CNCs)	12	208.1 ± 0.557	206.6–208.7	208.3 (208.0–208.4)			

*Note*:  ^∗^Statistically significant at *p* < 0.05.

Abbreviations: ANOVA, analysis of variance; NS, not significant.

**Table 2:** Pairwise comparison of wear resistance between study groups.

Group	Statistical measure	Control	G1 (0.2%CNCs)	G2 (0.5%CNCs)	G3 (1%CNCs)
Games–Howell post hoc test: wear resistance					
Control	Mean difference	—	18.2	18.0975	31.4
	*p*‐Value	—	**<0.001** ^∗^	**<0.001** ^∗^	**<0.001** ^∗^
G1 (0.2% CNCs)	Mean difference	—	—	−0.0925	13.2
	*p*‐Value	—	—	0.763	**<0.001** ^∗^
G2 (0.5% CNCs)	Mean difference	—	—	—	13.3
	*p*‐Value	—	—	—	**<0.001** ^∗^
G3 (1%CNCs)	Mean difference	—	—	—	—
	*p*‐Value	—	—	—	—

*Note:* Games–Howell post hoc test.  ^∗^Statistically significant at *p* < 0.05.

**Table 3:** Post hoc tests showing pair‐wise comparison of surface microhardness between study groups.

Group	Statistical measure	Control	G1 (0.2% CNCs)	G2 (0.5% CNCs)	G3 (1% CNCs)
Games‐Howell post hoc test: surface microhardness					
Control	Mean difference	—	−35.0	−59.0	−109.7
	*p*‐Value	—	**<0.001** ^∗^	**<0.001** ^∗^	**<0.001** ^∗^
G1 (0.2% CNCs)	Mean difference	—	—	−24.1	−74.8
	*p*‐Value	—	—	**<0.001** ^∗^	**<0.001** ^∗^
G2 (0.5% CNCs)	Mean difference	—	—	—	−50.7
	*p*‐Value	—	—	—	**<0.001** ^∗^
G3 (1% CNCs)	Mean difference	—	—	—	—
	*p*‐Value	—	—	—	—

*Note:* Games–Howell post hoc test.  ^∗^Statistically significant at *p* < 0.05.

**Figure 4:**
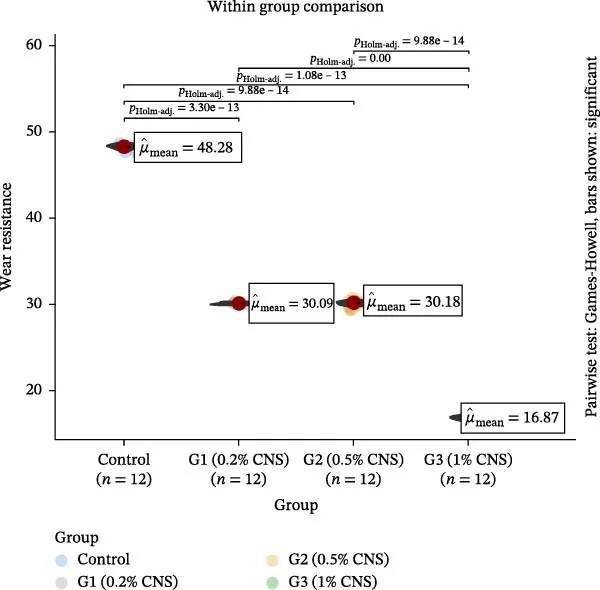
Distribution of wear resistance across study groups using violin plot analysis.

The mean wear resistance was highest in the control group (48.3 ± 0.310), followed by G2 (0.5% CNCs) (30.2 ± 0.281) and G1 (0.2% CNCs) (30.1 ± 0.167), while the lowest mean value was observed in G3 (1% CNCs) (16.9 ± 0.259). The median values showed a similar trend, with control recording 48.3 (48.1–48.5), G2 30.2 (30.0–30.3), G1 30.1 (29.9–30.2), and G3 16.9 (16.7–17.0). The range of values was narrow in all groups, indicating minimal variability within each experimental condition. One‐way ANOVA demonstrated a statistically highly significant difference in wear resistance among the four groups (*F* = 22,933, *p* < 0.001). On post hoc analysis using the Games–Howell test, the control group showed a statistically significantly higher wear resistance compared to G1, G2, and G3 (all *p* < 0.001). There was no statistically significant difference between G1 and G2 (mean difference = −0.0925, *p* = 0.763). (Tables 1 and 2). The distribution and spread of wear resistance across groups are illustrated in Figure 4.

## 4. Discussion

The present study demonstrated that CNCs incorporation significantly increased the surface microhardness of restorative GIC in a concentration‐dependent manner, with the highest microhardness observed in the 1% CNCs group. However, wear resistance showed an opposite trend, with higher CNCs concentrations exhibiting less favorable wear performance. These findings partially support the study hypothesis and were partly in line with previous studies [14–16], which reported an increase in mechanical properties following the addition of cellulose nanocrystals and cellulose nanofibers. The improvement may be attributed to the ability of CNCs to establish hydrogen bonding with the carboxylic groups of polyacrylic acid and hydroxyl groups of glass particles, thereby improving the matrix integrity and stress transfer within the cement structure [7–9].

The observed increase in surface microhardness is consistent with recent studies demonstrating enhanced mechanical properties of GICs reinforced with natural biopolymers, functionalized fibers [17–19], and bioactive additives such as bioactive glass, chitosan, phytochemicals, and nanoparticles [20–23]. Khan et al. [18] and Alsunbul et al. [17, 19] reported that oxidized biopolymers and functionalized glass fibers improved the mechanical properties of GIC through enhanced particle bonding and matrix cohesion. Unlike surface microhardness, wear resistance decreased with increasing CNCs concentration, with the highest wear observed in the 1% CNCs group. These findings are not in line with those of Silva et al. [6, 12], where fiber reinforcement has been linked with better abrasion resistance. A possible explanation is that excessive CNCs loading may lead to less uniform particle distribution within the matrix, adversely affecting wear resistance despite the observed increase in microhardness. Similar concentration‐dependent effects have been reported in reinforced GICs, where optimal filler concentrations produced superior mechanical properties [17–19]. Recent advances in GIC modification have increasingly focused on bioactive approaches that combine mechanical reinforcement with antimicrobial and remineralization potential [24–26]. Phytochemical additives, including plant‐derived extracts, such as *Citrus aurantium*, have demonstrated improvements in antibacterial activity while maintaining acceptable mechanical performance [27]. Similarly, green‐synthesized nanomaterials, including titanium dioxide nanoparticles, have shown enhanced hardness and flexural properties together with favorable biological safety profiles [24]. Supporting these findings, recent reviews [23, 25, 26] highlight that bioactive additives, nanoparticles, chitosan, and bioactive glass can enhance both biological and selected mechanical properties, although their effects remain formulation‐dependent and require further clinical validation. However, as wear resistance was not improved and declined at higher CNCs concentrations, direct clinical extrapolation should be approached with caution.

This in vitro study has several limitations. The experimental conditions did not fully replicate the oral environment, and thermocycling or artificial aging protocols were not performed. Additionally, only surface microhardness and wear resistance were evaluated, while other clinically relevant properties such as compressive strength, flexural strength, fracture toughness, sorption, and solubility were not assessed. Furthermore, advanced characterization techniques such as scanning electron microscopy (SEM), energy‐dispersive X‐ray spectroscopy (EDX), Fourier‐transform infrared spectroscopy (FTIR), and X‐ray diffraction (XRD) were not performed, limiting insights into CNCs dispersion and reinforcement mechanisms. Therefore, the generalizability of these findings to clinical conditions may be limited, and the results should be interpreted with caution when extrapolating to in vivo settings.

Future studies should incorporate aging protocols such as thermocycling, pH cycling, and fatigue loading, along with advanced characterization techniques (SEM, EDX, FTIR, and XRD) to better understand CNCs–GIC interactions. Evaluation of additional properties, including compressive strength, flexural strength, fracture toughness, sorption, solubility, fluoride release, and biocompatibility, is warranted. Further optimization of CNCs concentration and dispersion methods, followed by in vivo studies and clinical trials, is needed to establish the long‐term clinical effectiveness of CNCs‐modified GICs. Once proven, this material can be used in resource‐limited and community‐based setting where GIC is commonly used.

## 5. Conclusion

Within the limitations of this in vitro study, incorporation of cellulose nanocrystals significantly increased the surface microhardness of conventional restorative GIC in a concentration‐dependent manner. However, higher CNCs concentrations were associated with reduced wear resistance. Therefore, while CNCs demonstrate potential as reinforcing agents for enhancing surface hardness, this improvement alone does not necessarily translate into superior long‐term clinical performance, particularly in posterior load‐bearing restorations. Further long‐term in vivo studies and well‐designed clinical trials are required to validate these findings and establish the material’s overall clinical suitability under oral conditions.

## Author Contributions

**Kiran Manjunath:** conceptualization, methodology, investigation, data curation, formal analysis, writing – original draft preparation. **Vinod Jathanna:** supervision, validation, writing – review and editing. **Kishore Ginjupalli:** methodology, formal analysis, resources, validation. **Shreya Hegde:** conceptualization, supervision, project administration, validation, writing – review and editing, corresponding author responsibilities.

## Funding

The authors declare that no financial support was received for the research and/or publication of this article. This work did not receive any specific grant from funding agencies in the public, commercial, or not‐for‐profit sectors.

## Disclosure

The presentation of this study is done by the first author in the 29th IAPHD National Conference 2025, Mangaluru. All authors have read and approved the final version of the manuscript. The corresponding author had full access to all of the data in this study and takes complete responsibility for the integrity of the data and the accuracy of the data analysis. All content generated or refined with the assistance of ChatGPT was critically reviewed, verified, and approved by the authors, who take full responsibility for the integrity, accuracy, and originality of the manuscript.

## Conflicts of Interest

The authors declare no conflicts of interest.

## Data Availability

This article is available from the first author upon request.
